# Effects of High Intensity Interval Training on Pregnant Rats, and the Placenta, Heart and Liver of Their Fetuses

**DOI:** 10.1371/journal.pone.0143095

**Published:** 2015-11-13

**Authors:** Nils Thomas Songstad, Knut-Helge Frostmo Kaspersen, Anne Dragøy Hafstad, Purusotam Basnet, Kirsti Ytrehus, Ganesh Acharya

**Affiliations:** 1 Women’s Health and Perinatology Research Group, Department of Clinical Medicine, Faculty of Health Sciences, UiT–The Arctic University of Norway, Tromsø, Norway; 2 Department of Pediatrics, University Hospital of Northern Norway, Tromsø, Norway; 3 Pediatric Research Group, Department of Clinical Medicine, Faculty of Health Sciences, UiT–The Arctic University of Norway, Tromsø, Norway; 4 Cardiovascular Research Group, Department of Medical Biology, Faculty of Health Science, UiT–The Arctic University of Norway, Tromsø, Norway; 5 Department of Obstetrics and Gynecology, University Hospital of Northern Norway, Tromsø, Norway; Max-Delbrück Center for Molecular Medicine (MDC), GERMANY

## Abstract

**Objective:**

To investigate the effects of high intensity interval training (HIIT) on the maternal heart, fetuses and placentas of pregnant rats.

**Methods:**

Female Sprague-Dawley rats were randomly assigned to HIIT or sedentary control groups. The HIIT group was trained for 6 weeks with 10 bouts of high intensity uphill running on a treadmill for four minutes (at 85–90% of maximal oxygen consumption) for five days/week. After three weeks of HIIT, rats were mated. After six weeks (gestational day 20 in pregnant rats), echocardiography was performed to evaluate maternal cardiac function. Real-time PCR was performed for the quantification of gene expression, and oxidative stress and total antioxidant capacity was assessed in the tissue samples.

**Results:**

Maternal heart weight and systolic function were not affected by HIIT or pregnancy. In the maternal heart, expression of 11 of 22 genes related to cardiac remodeling was influenced by pregnancy but none by HIIT. Litter size, fetal weight and placental weight were not affected by HIIT. Total antioxidant capacity, malondialdehyde content, peroxidase and superoxide dismutase activity measured in the placenta, fetal heart and liver were not influenced by HIIT. HIIT reduced the expression of eNOS (p = 0.03), hypoxia-inducible factor 1α (p = 0.04) and glutathione peroxidase 4.2 (p = 0.02) in the fetal liver and increased the expression of vascular endothelial growth factor-β (p = 0.014), superoxide dismutase 1 (p = 0.001) and tissue inhibitor of metallopeptidase 3 (p = 0.049) in the fetal heart.

**Conclusions:**

Maternal cardiac function and gene expression was not affected by HIIT. Although HIIT did not affect fetal growth, level of oxidative stress and total antioxidant capacity in the fetal tissues, some genes related to oxidative stress were altered in the fetal heart and liver indicating that protective mechanisms may be activated.

## Introduction

Clinical guidelines encourage moderate exercise in pregnancy due to its multiple beneficial effects for both the mother and her offspring [1–3]. In long-term, women who continue to exercise during pregnancy appear to exercise at a higher intensity, deposit less body fat, improve fitness and have a lower cardiovascular risk profile than those who cease to exercise during pregnancy [4]. There is some evidence that physical exercise during pregnancy might mitigate the effects of placental insufficiency or the angiogenic imbalance associated with preeclampsia [5,6]. Regular exercise during pregnancy reduces the risk of having a large for gestation newborn, without a change in risk of having a small for gestation newborn [7], does not negatively affect physical growth or neurodevelopmental outcome at five years and may reduce subcutaneous fat mass in offspring [8]. In pregnant well-trained athletes, exercising for 8 hours per week (range, 3 to 13.5) does not appear to pose any health risk and maximal oxygen consumption ($\dot{\text{V}}$O_2max_) is maintained throughout pregnancy and post-partum [9]. However, there remains some concern regarding high intensity training because of possible adverse effect on placental blood flow [10], and guidelines on high intensity training during pregnancy are conflicting [11].

High-intensity interval training (HIIT) consists of regular bouts of strenuous exercise at the anaerobic threshold and in healthy individuals it may be a more efficient method of improving maximal oxygen consumption ($\dot{\text{V}}$O_2max_) than continuous workout at 70% or 85% of maximal heart rate [12,13]. HIIT is gaining popularity as a time-efficient exercise strategy for improving health and fitness, and regularly exercising women may wish to continue HIIT in pregnancy. Furthermore, obesity is a known risk factor for adverse pregnancy outcomes [14] and HIIT may be an effective way of reducing weight [15,16]. Thus, HIIT may be beneficial in pregnancies complicated by obesity. However, the safety and efficacy of HIIT during pregnancy is not known, and to our knowledge the effects of HIIT on the maternal heart, $\dot{\text{V}}$O_2max_, placenta or fetus have not been studied in pregnant women or animal models previously.

Our main objective was to investigate if HIIT started three weeks before pregnancy and continued close to term alters maternal cardiac structure, function, and gene expression and to determine if HIIT has any adverse effects on the fetus in a rat model.

## Materials and Methods

Animal experiments conformed to the Directive 2010/63/EU of the European Parliament on the protection of animals used for scientific purposes [17] and all procedures were approved by the Norwegian Committee on Ethics in Animal Experimentation (project ID 2853).

### Design of study and training of rats

Forty-eight young (9–11 weeks old) female Sprague-Dawley rats (Charles River, Sulzfeld, Germany) weighing 233±3g at inclusion were randomly assigned to HIIT or a sedentary control group. As rats are more active at night and the experiments were performed at daytime, the circadian rhythm of the rats was changed by reversing light/dark (12/12 hour) cycle, and the rats were allowed at least 5 days of acclimation. All rats had free access to tap water and were fed a pellet diet especially produced for breeding rodents (Rat and Mouse NO.3 Breeding, Special Diet Services, Witham, Essex, U.K.) *ad libitum*.

**Fig 1:**
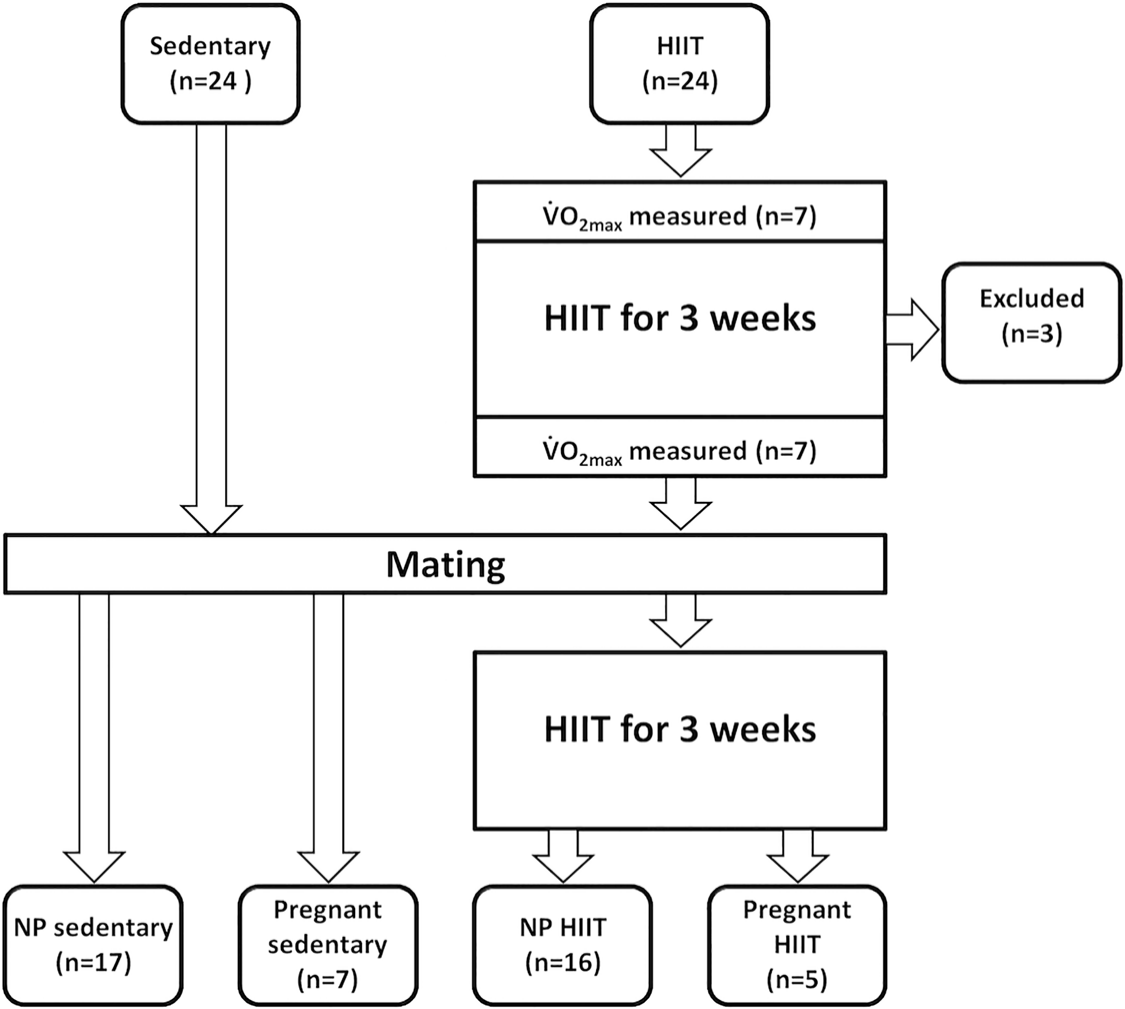
Study design. Flow chart illustrating the design of the study. HIIT, high intensity interval training, $\dot{\text{V}}$O_2max_, maximal oxygen consumption, NP, non-pregnant.

The design of the study is summarized in Fig 1. In HIIT rats, $\dot{\text{V}}$O_2max_ was measured using a treadmill at 25° inclination in a metabolic chamber (Modular treadmill with Oxymax open circuit calorimeter, Columbus Instruments, OH, USA) and was calculated at the start and after three weeks of HIIT in seven animals. The speed was gradually increased until oxygen consumption leveled off despite increased running speed, and $\dot{\text{V}}$O_2max_ was defined as $\dot{\text{V}}$O_2_ measured at this speed [18]. These $\dot{\text{V}}$O_2max_ values were used to set the speed of the treadmill for HIIT.

For the HIIT exercise protocol (five days/week), rats were running for 5 minutes at low speed (at <60% of $\dot{\text{V}}$O_2max_) for warm up, and then subjected to exercise sessions of 10 bouts of four minutes high intensity running on a treadmill at 25° inclination (running at 85–90% of $\dot{\text{V}}$O_2max_) alternating with two minutes of active recovery (running at 50–60% of $\dot{\text{V}}$O_2max_). Three or four rats ran in parallel tracks on the same treadmill. Stimulation such as gentle physical handling, an airbrush or low current shock grids were used to secure high intensity, but kept to a minimum. Typically rats tolerated and achieved highest speed in the last bouts of each session. All rats were continuously monitored during the training sessions. Throughout the training period treadmill speed was increased gradually as long as the rats did not show signs of exhaustion, aiming for the anaerobic threshold at approximately 85–90% of $\dot{\text{V}}$O_2max_. Treadmill inclination was kept at 25°. Rats assigned to the sedentary control group were not exposed to treadmill running.

After three weeks of HIIT $\dot{\text{V}}$O_2max_ was measured again in seven rats (Fig 1). Training was stopped for one day and one male rat was put in a cage with two female rats for 6–7 hours for mating. In pregnant rats, the day of mating was considered gestational day (GD) 0. Two days after mating, the rats resumed HIIT again without the knowledge of pregnancy status. Each rat completed a total of 24 training sessions of 63 minutes of running on the treadmill per session with a total running time of 1512 minutes per rat. Considering the welfare of pregnant rats close to term, the training was stopped 2–3 days prior to terminal experiments. Sedentary control rats of same age and size were housed in cages in the same rooms as HIIT rats, and were not exposed to treadmill training.

### Terminal experiments

Twenty days after mating (GD20 in pregnant rats), M-mode echocardiography was performed using a Vevo-770 system (VisualSonics, Toronto, Canada) as described previously [19,20]. In short, the rats were anesthetized with isoflurane (Vevo Compact Anesthesia System, VisualSonics, Toronto, Canada), intubated and ventilated (New England Medical Instruments Inc., Medway, MA, USA) before echocardiography was performed. The heart rate was obtained from the electro-cardiogram signals. Ultrasound measurements were performed off-line without the knowledge of the animals’ training and pregnancy status. Left ventricular fractional shortening, ejection fraction and stroke volume were calculated from M-mode echocardiography [20], and cardiac output was calculated as; stroke volume x heart rate. The animals were euthanized with sodium pentobarbital 100 mg/kg. Pregnancy status was determined at autopsy. The heart, left ventricle (LV), fetuses and placentas were weighed and tibia length and fetal crown-rump length (CRL) were measured. Heart weight, LV weight and heart weight/tibia length ratio were used as parameters of heart hypertrophy as heart weight/body weight ratio is not applicable due to physiological weight gain in pregnancy [20]. Placental efficiency was calculated as a ratio between fetal and placental weight [21]. Tissue samples collected from the maternal LV, placentas, fetal hearts and fetal livers were stored for the measurement of oxidative stress, total antioxidant capacity and gene expression analyses using quantitative RT-PCR. All analyses were performed in a blinded fashion without the knowledge of training and pregnancy status.

### Measurement of oxidative stress and total antioxidant capacity

Frozen tissue samples from 2–3 feto-placental units from each dam were thawed, weighed and homogenized for 2–3 min with a mechanical homogenizer in the buffer supplied in the assay kits to prepare 50 mg/ml suspensions. The homogenized suspension was centrifuged to 14000 x g for 15 minutes at 4°C. The supernatant was separated and stored in working aliquots at -70°C. All the analyses were performed on the supernatant according to the instructions provided by the manufacturers of the assay kits. *Malondialdehyde (MDA) content* was quantified by using OxiSelectTM TBARS Assay kit (Cell Biolabs, Inc., San Diego, CA, USA). All samples and standards were assayed in duplicate. The thiobarbituric acid reaction was completed in a microcentrifuge tube (1.5 mL) at 95° C for 1 hour. 200 μL of the reaction product was transferred into a 96 well microplate and the colour was read with a spectrophotometric plate reader at 532 nm using a blank as control. The *superoxide dismutase (SOD) activity* kit (Abnova GmbH EMBLEM, Heidelberg, Germany) measures total (i.e. cytosolic and mitochondrial) SOD activity. All reactions were carried out in a 96 well microplate in triplicate, and the absorbance was read at 450 nm. *Peroxidase activity* reactions (Sigma-Aldrich, St. Louis, MO, USA) were carried out in a 96 well microplate in quadruplet, and the absorbance was read at 570 nm. Cumulative *antioxidant capacity* was quantified using an antioxidant assay kit (Sigma-Aldrich, St. Louis, MO, USA). All reactions were carried out in a 96 well microplate in duplicate. The absorbance was read at 405 nm and the results are presented as the inverse of optical density readings.

### Quantitative RT-PCR

RT-PCR was performed as described previously [19]. The expression of the target genes were normalized to the stably expressed reference genes. Twenty-two genes with potential relation to cardiac remodeling were examined in maternal LV myocardium. Expression of genes related to oxidative stress and cardiac remodelling was examined in placentas (11 genes), fetal livers (13 genes) and fetal hearts (15 genes). A total of 28 feto-placental units (2–3 from each mother) were examined and the mean value for each mother was calculated. In addition, expression of Ddx3y (DEAD box polypeptide 3, Y-linked) and Eif2s3y (eukaryotic translation initiation factor 2, subunit 3, Y-linked) genes were analyzed in fetal hearts to differentiate male from female fetuses. Expression of genes was normalized to mean values of sedentary non-pregnant (maternal heart) or sedentary pregnant rats (placenta, fetal heart and fetal liver). The reference genes and primers used are available as supporting information (S1 Table).

### Statistical analyses

All data are reported as mean ± standard error of the mean (SEM). Mean values from each mother are used when comparing fetal data. A p-value <0.05 was considered statistically significant. Logarithmic transformation was used to achieve normal distribution of continuous variables when appropriate. One way ANOVA was used to compare the four groups of rats: Non-pregnant HIIT, pregnant HIIT, non-pregnant sedentary and pregnant sedentary. Two way ANOVA was used to investigate the influence and interaction between pregnancy status and HIIT on gene-expression data. The Holm–Sidak method was used as post-hoc test. Independent-Samples T-test was used to compare fetal outcome from HIIT and sedentary pregnant rats. Chi-square test was used to compare groups when using categorical variables. PASW Statistics 18.0.3 (SPSS Inc., Chigaco, IL, USA) and Sigma Plot 12.0 (Systat Software Inc, San Jose, CA, USA) softwares were used for statistical analyses.

## Results

Twenty-four female rats were subjected to HIIT and 24 were used as sedentary controls (Fig 1). Three rats sustained tail injures during exercise on the treadmill and were excluded before mating. The pregnancy rate was 5/20 mated rats in the group subjected to HIIT and 7/20 in the sedentary group (p = 0.7). Five control rats were not mated. Thus a total of 45 animals were included in the final analysis; 5 pregnant HIIT, 7 pregnant sedentary, 16 non-pregnant HIIT and 17 non-pregnant sedentary.

All rats tolerated HIIT, but the amount of stimulation necessary to keep the rats running at the required pace was individual. There were no significant differences in running speed or total running distance between pregnant and non-pregnant rats (Table 1). $\dot{\text{V}}$O_2max_ was calculated at baseline and before mating in the first seven rats. Calculated $\dot{\text{V}}$O_2max_ did not change after three weeks of HIIT (78±3 versus 75±4 mL/kg^/^min, p = 0.6), but the treadmill speed at $\dot{\text{V}}$O_2max_ was increased from 18.6±0.5 to 21.9±1.3 m/min (p = 0.006).

**Table 1:** Characteristics of study groups.

	NP sedentary	NP HIIT	Pregnant sedentary	Pregnant HIIT
Number of animals	17	16	7	5
Average speed at intervals 2^nd^ week (m/min)	-	15.2±0.6	-	15.3±0.5
Average speed at intervals 6^th^ week (m/min)	-	21.2±1.2	-	22.4±2.0
Total running distance (km)	-	82±5	-	85±6
Body weight (g)	272±7	269±4	372±9^*†^	360±14^*†^
Total heart weight (g)	0.78±0.02	0.82±0.02	0.79±0.02	0.79±0.03
Left ventricular weight (g)	0.55±0.01	0.57±0.01	0.55±0.01	0.55±0.03
Heart weight/tibia ratio (mg/mm)	20.0±0.6	20.4±0.5	20.1±0.4	20.1±0.7
Heart rate	385±8	378±16	364±16	386±17
Ejection fraction (%)	70±2	65±3	77±3	75±5
Cardiac output (mL/min)	72±4	66±5	75±8	74±9
Number of foetuses pr litter	-	-	14±1	13±2
Foetal weight (g)	-	-	2.8±0.1	2.8±0.2
CRL (mm)	-	-	32±0	33±1
Foetal heart weight (mg)	-	-	28±1	26±1
Placenta weight (g)	-	-	0.52±0.04	0.54±0.05
Placental efficiency	-	-	5.5±0.5	5.5±0.7

Data presented as mean±SEM. Independent-Samples T-test was used to compare two groups. Comparisons between four groups were made using one-way ANOVA and Holm-Sidak post hoc test. Placental efficiency, ratio between foetal and placental weight.

NP, non-pregnant. HIIT, High intensity interval training. CRL, crown-rump-length.

p<0.05 compared to NP sedentary (*) and NP HIIT (†).

Body weight was increased by pregnancy, but not affected by exercise in pregnant or non-pregnant rats (Table 1). There were no differences in resting heart rate, cardiac output, heart weight, LV weight and heart weight/tibia length ratio, between any groups (Table 1). There was no correlation between total distances ran and heart or LV weight in HIIT rats. Weight gain in pregnancy was not significantly different in sedentary and HIIT rats (123±12 versus 111±8 g, p = 0.4). Expressions of genes related to cardiac remodeling in the maternal heart and the influence by pregnancy independent of HIIT is shown in Table 2. None of the genes examined were influenced by HIIT independent of pregnancy.

**Table 2:** Maternal myocardial expression of genes related to cardiac remodeling in pregnant and non-pregnant HIIT compared to pregnant and non-pregnant sedentary rats and influence of pregnancy on gene expression.

Gene	NP sedentary (n = 7)	NP HIIT (n = 7)	Pregnant sedentary (n = 7)	Pregnant HIIT (n = 5)	Influence of pregnancy
**Heart function**					
PKCα	1.00±0.06	1.05±0.10	0.89±0.07	1.06±0.08	-
PKCδ	1.00±0.11	0.95±0.08	0.76±0.05	0.81±0.07	↓
PKCε	1.00±0.05	1.01±0.09	1.07±0.04	1.10±0.09	-
α-MHC	1.00±0.02	0.88±0.02	0.80±0.04^*^	0.84±0.05^*^	na
β-MHC	1.00±0.09	1.91±0.36	3.72±0.83^*^	1.29±0.28^#^	-
ANP	1.00±0.35	1.32±0.28	0.35±0.06^§^	0.37±0.08	↓↓
BNP	1.00±0.14	1.78±0.12^*^	0.39±0.10^*§^	0.49±0.12^§^	na
ANKRD1	1.00±0.07	1.12±0.08	0.55±0.05^*§^	0.58±0.06^*§^	↓↓
TNFα	1.00±0.12	0.93±0.16	1.03±0.13	0.75±0.07	-
TGFβ1	1.00±0.04	0.95±0.03	0.83±0.04^*^	0.90±0.07	↓
TGFβ2	1.00±0.10	1.10±0.13	0.78±0.06	0.81±0.05	↓
TGFβ3	1.00±0.07	0.87±0.05	0.79±0.02^*^	0.74±0.05^*^	↓
CTH	1.00±0.17	0.77±0.14	0.88±0.09	0.59±0.10	-
**Angiogenesis**					
VEGF-α	1.00±0.09	1.08±0.09	0.89±0.07	1.06±0.09	-
VEGF-β	1.00±0.03	0.97±0.04	0.85±0.03^*^	0.88±0.05	↓
**Oxidative stress**					
SOD 1	1.00±0.05	1.15±0.06	0.96±0.08	1.03±0.09	-
eNOS	1.00±0.12	0.94±0.05	0.88±0.07	0.79±0.07	-
iNOS	1.00±0.18	1.13±0.12	0.61±0.05^§^	0.58±0.12	↓
**Fibrosis**					
COL1A1	1.00±0.13	0.84±0.13	0.69±0.06	0.65±0.12	↓
COL3A1	1.00±0.14	0.84±0.13	0.71±0.06	0.56±0.10	↓
FN1	1.00±0.10	0.77±0.07	0.62±0.06^*^	0.77±0.09	na
TIMP1	1.00±0.06	0.76±0.07	0.85±0.05	0.78±0.20	↓

Relative expression of genes normalized to mean values in NP sedentary rats. Data are presented as mean±SEM.

Comparisons between groups were made using one-way ANOVA and influence of pregnancy was analyzed using two-way ANOVA. Holm-Sidak post hoc test was used.

NP, non-pregnant, HIIT, high intensity interval training, PKC, protein kinase C, MHC, myosin heavy chain, ANP, atrial natriuretic peptide, BNP, B-type natriuretic peptide, ANKRD1, ankyrin repeat domain-containing protein 1, TNF-α, tumor necrosis factor-α, TGF, transforming growth factor, CTH, cystathionase, VEGF, vascular endothelial growth factor, SOD, superoxide dismutase, eNOS, endothelial nitric oxide synthase, iNOS, inducible nitric oxide synthase, COL1A1, collagen type I-α1, COL3A1, collagen type III-α1, FN1, fibronectin 1, TIMP1, tissue inhibitor of metallopeptidase 1

p<0.05 compared to non-pregnant sedentary (*), non-pregnant HIIT (§), or pregnant sedentary rats (#), ↓ decrease p<0.05, ↓↓ decrease p<0.001, -, non-significant influence, na, non-applicable due to interaction.

**Fig 2:**
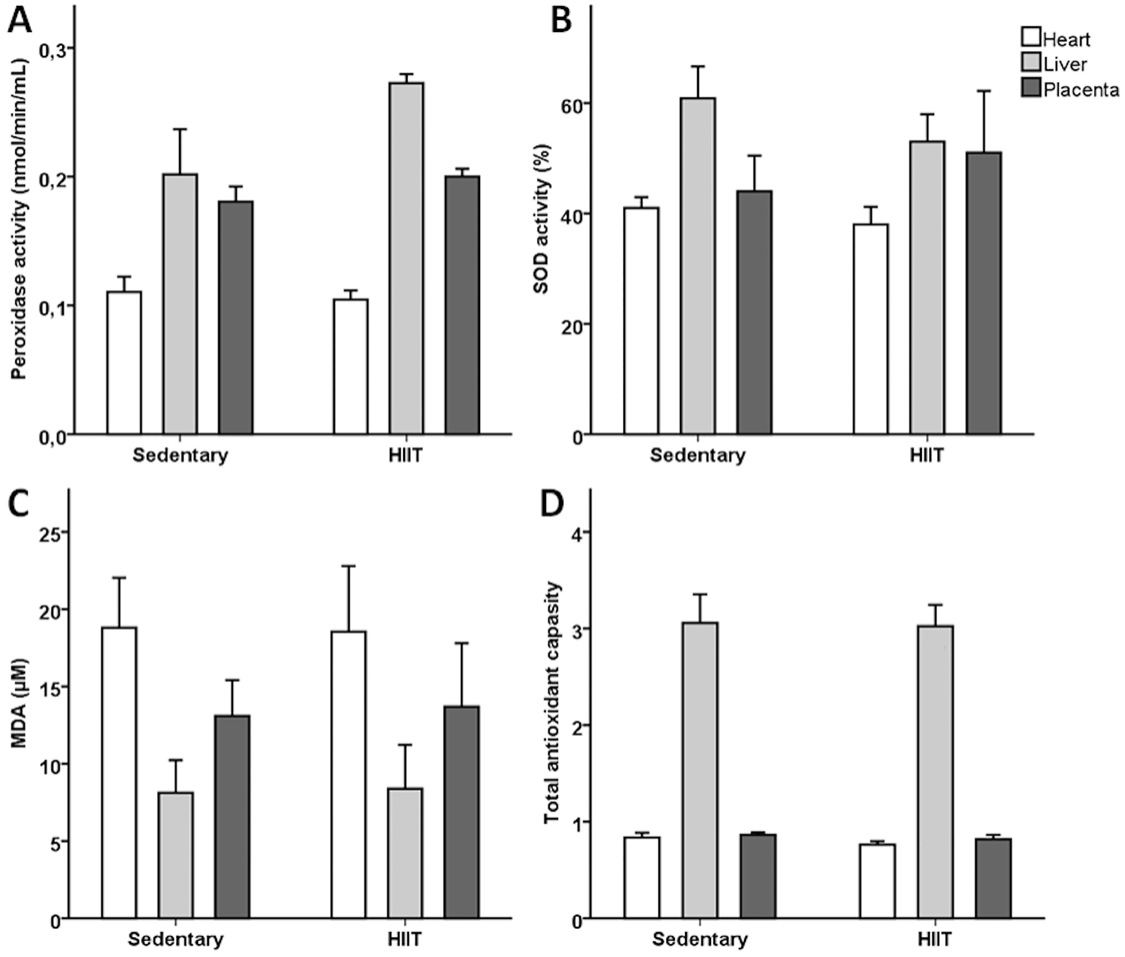
Total antioxidant capacity and oxidative stress level in placenta and fetal tissues. Peroxidase activity (A), superoxide dismutase (SOD) activity (B), malondialdehyde (MDA) content (C) and total antioxidant capacity presented as the inverse of optical density readings (D) in fetal hearts, fetal livers and placentas form high intensity interval training (HIIT, n = 5) and sedentary rats (n = 7). Data are presented as mean±SEM.

There were no differences in litter size, fetal weight, CRL, placenta weight, placental efficiency or fetal heart weight between exercised and sedentary pregnant rats (Table 1). The uterus was inspected in all rats and there were no signs of previous resorption of pregnancy in any of the non-pregnant rats. HIIT did not significantly affect total antioxidant capacity or oxidative stress level in placenta, fetal liver or fetal heart (Fig 2). In both groups, total antioxidant capacity was higher in fetal liver tissue (3.04±0.19) compared to fetal heart (0.81±0.03, p<0.001) and placenta (0.84±0.02, p<0.001). Expression of genes related to cardiac remodeling and oxidative stress in the fetal heart, fetal liver and placenta is presented in Table 3. Fetal sex did not affect the expression of any of the selected genes (data not shown). Expression of SOD1 (p = 0.001), VEGF-B (p = 0.014) and TIMP3 (p = 0.049) was increased in the fetal hearts in HIIT rats. Expression of eNOS (p = 0.03), HIF-1A (p = 0.04) and GPx4.2 (p = 0.02) was reduced by HIIT in fetal liver whereas in placenta no examined genes were significantly affected.

**Table 3:** Fetal and placental gene-expression.

	Fetal heart		Fetal liver		Placenta	
Gene expression	Sedentary (n = 7)	HIIT (n = 5)	Sedentary (n = 7)	HIIT (n = 5)	Sedentary (n = 7)	HIIT (n = 5)
PKCα	1.00±0.07	1.26±0.10	-	-	-	-
PKCδ	1.00±0.03	1.05±0.03	-	-	-	-
PKCε	1.00±0.02	1.07±0.03	-	-	-	-
α-MHC	1.00±0.13	1.07±0.13	-	-	-	-
β-MHC	1.00±0.02	0.98±0.05	-	-	-	-
ANP	1.00±0.13	1.24±0.16	-	-	-	-
BNP	1.00±0.10	1.16±0.11	-	-	-	-
VEGF-A	1.00±0.03	1.08±0.04	1.00±0.08	0.93±0.10	1.00±0.09	1.05±0.06
VEGF-B	1.00±0.02	1.12±0.04*	1.00±0.06	0.92±0.03	1.00±0.18	0.80±0.21
SOD1	1.00±0.03	1.21±0.03**	1.00±0.04	1.16±0.0.15	1.00±0.06	1.01±0.09
SOD2	1.00±0.02	1.0 5±0.03	1.00±0.03	1.10±0.14	1.00±0.06	0.99±0.04
eNOS	-	-	1.00±0.02	0.84±0.07*	1.00±0.18	1.03±0.19
iNOS	1.00±0.04	0.84±0.08	1.00±0.20	1.17±0.31	1.00±0.11	0.81±0.08
TIMP1	1.00±0.03	1.02±0.07	-	-	-	-
TIMP3	1.00±0.06	1.15±0.02*	-	-	-	-
TIMP4	1.00±0.05	1.02±0.02	-	-	-	-
HIF1A	-	-	1.00±0.07	0.76±0.07*	1.00±0.06	1.03±0.07
CAT	-	-	1.00±0.07	0.92±0.08	1.00±0.10	1.29±0.18
HK2	-	-	1.00±0.11	1.06±0.16	1.00±0.08	1.25±0.12
GPx1	-	-	1.00±0.05	0.87±0.03	1.00±0.10	0.93±0.10
GPx2	-	-	1.00±0.15	0.74±014	1.00±0.10	0.87±0.08
GPx4.1	-	-	1.01±0.08	0.81±0.08	-	-
GPx4.2	-	-	1.01±0.12	0.57±0.07*	-	-

Relative expression of genes in fetal heart, fetal liver and placenta normalized to mean values in sedentary rats. Data are presented as mean±SEM. Independent-Samples T-test was used to compare two groups. PKC, protein kinase C, MHC, myosin heavy chain, ANP, atrial natriuretic peptide, BNP, brain natriuretic peptide, VEGF, vascular endothelial growth factor, SOD, superoxide dismutase, eNOS, endothelial nitric oxide synthase, iNOS, inducible nitric oxide synthase, TIMP, tissue inhibitor of metallopeptidase, HIF1A, hypoxia-inducible factor 1α, CAT, catalase, HK2, hexokinase II, GPx, glutathione peroxidase.

*, p<0.05

**, p<0.001

## Discussion

Our study shows that HIIT is well tolerated by pregnant rats. Despite an increase in treadmill running speed indicative of training adaptation, six weeks of HIIT did not lead to significant changes in cardiac structure, function, and gene expression in young female rats whether they became pregnant or not. HIIT throughout the pregnancy did not affect the size of placentas or fetuses and did not induce significant oxidative stress in placenta, fetal liver or heart. However, the expression of some genes related to oxidative stress was found to be altered in fetal liver and heart.

### Effects of pregnancy and HIIT on the mother

HIIT rats were able to increase their maximal running speed at intervals by 42% during the training period, indicating that their physical fitness improved. As this increase in speed was similar both in pregnant and non-pregnant rats, it is unlikely to be a result of physiological changes related to pregnancy. However, HIIT for six weeks did not lead to heart hypertrophy irrespective of pregnancy status. This is in contrast to *Wisløff et al* [18] who reported a significant increase in LV mass of ~10% in female Sprague-Dawley rats after four weeks of HIIT and a ~35% increase after 13 weeks. In their study the rats exercised for a longer time period (2 h/day) at intervals of 8 min duration at 85–90% of $\dot{\text{V}}$O_2max_ [18]. It is possible that longer training sessions or longer intervals would have increased LV mass. However, HIIT protocols similar to ours have been used in non-pregnant rats [22] and mice [23] previously. A longer total period of HIIT was not applicable due to the short duration of pregnancy in rats (~21 days). As our goal was to investigate the effect of HIIT in pregnancy we did not consider extending the training period before mating.

Despite the fact that the HIIT rats completed a total of 24 one-hour sessions of HIIT with a total running distance of more than 80 kilometers, significant heart hypertrophy was not noted. We used young (9–11 weeks at inclusion) female rats that continued to grow during the training period, and the hearts may not have reached adult size at inclusion. Thus we speculate that the heart of maturing young female rats may respond to the physiologic stimuli of pregnancy and/or exercise differently than the heart of mature adult rats. Furthermore, HIIT did not alter LV ejection fraction in both non-pregnant and pregnant rats (Table 1). Interestingly, expression of none of the 22 examined genes related to cardiac function and remodeling was significantly affected by HIIT. However, expression of 11 of these genes was influenced by pregnancy (Table 2), in line with our findings in pregnant Wistar rats [19,20]. Contrary to what is seen in humans [24] and mice [25], pregnancy *per se* did not increase LV mass in rats. This confirms the findings of previous studies in young rats [19,20,26,27].

### Effect on the placenta and fetus

Vigorous training during pregnancy does not appear to affect fetal wellbeing or offspring negatively [8,9]. However, exercise at the anaerobic threshold (more than 90% of maximal maternal heart rate) has been shown to cause a 50% reduction in uterine artery volume blood flow, a significant increase in umbilical artery pulsatility index and fetal bradycardia in a study on elite athletes in the second trimester of pregnancy [10]. During HIIT, exercise is performed at the anaerobic threshold for short intervals. Furthermore, in some previous studies on pregnant rats, forced and continuous high intensity training lead to impaired fetal growth [28,29]. However, in the present study there were no indications of adverse effect on fetal growth. This may indicate that the regular short periods of active recovery between bouts of exercise at the anaerobic threshold in HIIT, may protect the fetus and placenta from circulatory stress.

Decreased total antioxidant capacity and increased level of MDA together with the decreased SOD and peroxidase activities are indicators of oxidative stress [30]. We found no significant difference in the level of oxidative stress in the placenta and fetal tissues of HIIT rats compared to controls (Fig 2). Furthermore, HIIT did not alter genes related to oxidative stress in placenta. *Ramírez-Vélez et al* have reported a significant increase in eNOS expression and nitric oxide production and a decrease in hydrogen peroxide and mitochondrial superoxide levels in the placentas of women training at 55–75% of maximum heart rate three times a week during pregnancy compared to controls [31]. Although species differences could explain this discrepancy, the findings are likely to be related to the fact that the human placentas were collected after labour, which is shown to induce placental oxidative stress [32].

HIIT led to a significant increase in expression of SOD1, VEGF-B and TIMP3 in fetal hearts, and a decreased expression of eNOS, HIF-1A and GPx4.2 in fetal livers. The superoxide dismutases protect from damage caused by free radicals by catalyzing the conversion of the superoxide radical into hydrogen peroxide and oxygen [33]. SOD1 is present in the mouse heart during embryogenesis [34], and may have a role in protecting the fetal heart from oxidative stress. Hypoxia-inducible factors play key roles in the physiological response to hypoxia both through stabilization of proteins and regulation of gene expression [35], and the expression of HIF-1A in fetal liver is reduced by hypoxia [36]. eNOS is believed to play a key role in regulating the vascular tone in the normal liver [37], and the expression and activity of eNOS in the rat liver increases during late fetal life and peaks at 20 days of age in the rat pups [38]. GPx4 is an antioxidant enzyme witch catalyses peroxides including lipid hydroxyperoxide and its presence is vital for embryonic development [39]. Alteration of these genes observed in the fetal heart and liver could be a protective mechanism in response to hypoxic stress caused by HIIT. Furthermore, we found significantly (p<0.001) lower levels of total antioxidant capacity in the fetal heart and placenta compared to fetal liver in both groups of rats (Fig 2). This may indicate that placenta and fetal heart are more vulnerable than liver to oxidative stress.

### Limitations

One of the limitations of our study is that six weeks of HIIT followed by a two-days resting period did not lead to significant changes in selected markers of adaptation in the hearts of pregnant or non-pregnant rats. Due to animal welfare regulations HIIT was stopped 2–3 days before terminal experiments were performed on GD20. It is unlikely that significant changes in cardiac structure or function attributed to HIIT would disappear after a few days of inactivity. However, it could be argued that altered gene expression caused by HIIT could have been damped by 2–3 days of inactivity before tissues were sampled [40]. To avoid this, it may be advisable to perform terminal experiments and tissue sampling before HIIT is stopped. We chose to perform terminal experiments at term to be able to properly assess the fetal outcome, as significant fetal growth occurs during the last few days of pregnancy in rats [41]. Furthermore, considering the translational aspect of the study, we believe most pregnant women would refrain from HIIT in the last part of the third trimester. Body weight was not altered by HIIT. This is in line with the findings in previous studies on HIIT in non-pregnant non-obese rats [18] and does not indicate that the training was not effective. As our primary objective was to study the effect of HIIT on the maternal heart and the fetus, maternal skeletal muscle, fatty tissue or liver were not analyzed. Examinations of these organs could have strengthened the study.

In non-pregnant rats, *Wisløff et al* [18] have demonstrated the feasibility of measuring $\dot{\text{V}}$O_2max_ before and during the HIIT to assess the efficiency of training. Ideally, to ensure that the rats are running at their anaerobic threshold, it is recommended to do new $\dot{\text{V}}$O_2max_ measurements at regular time intervals in all rats [42,43]. However, in the present study $\dot{\text{V}}$O_2max_ was measured only in the first seven rats. Calculated $\dot{\text{V}}$O_2max_ did not increase after three weeks of HIIT, in spite of the rats being able to run significantly faster. We believe this illustrates the limitations of $\dot{\text{V}}$O_2max_ measurement in rats and thus out of animal welfare considerations $\dot{\text{V}}$O_2max_ measurements were stopped after the first group of animals, and running speed in each session was set by the best judgment of the operator.

Forced training may increase corticosteroid levels in pregnant rats whereas voluntary training does not [44]. In the present study the use of physical force to stimulate the rats to run on the treadmill was kept to an absolute minimum. However, a physiological stress response in the pregnant HIIT animals that might have contributed to the changes in gene expressions found in fetuses and placentas cannot be ruled out. Therefore, measurements of corticosteroids and other stress hormones should be considered in future studies on HIIT using pregnant rats.

The pregnancy rate following mating was low in the present study compared to that in our previous studies [19,20]. As there were no significant differences in pregnancy rates between HIIT and sedentary rats, it could be due to a shorter mating time (6–7 hours versus 12–18 hours), reversed light/dark cycle or differences between strains (Sprague-Dawley versus Wistar). The small number of pregnant HIIT rats (n = 5) limits our study’s ability to detect minor differences between groups.

### Translational perspective

HIIT did not affect body weight significantly in non-pregnant or pregnant rats and all rats gained weight during the course of experiments. This suggests that HIIT is not effective in reducing weight in young female non-obese rats, independent of pregnancy status, and this is in line with previous studies of non-pregnant female rats [18]. Maternal obesity and excessive weight gain in pregnancy are known risk factors for adverse pregnancy outcomes [14,45]. As the prevalence of obesity in pregnancy is increasing [45], from a translational perspective it would be of interest to examine the effects of HIIT in pregnant obese animals. It is possible that the ratio of skeletal muscle to adipose tissue mass is affected by training. Measures of body composition and other markers of metabolic profile could be addressed in future studies on HIIT in pregnancy.

The present study was performed in young, healthy rats. In the developed world the average maternal age is rising [46] and in future studies we would consider replacing young, adolescent rats with fully mature rats.

Prenatal hypoxic episodes could affect fetal development [30] and neonatal outcome [47]. We observed a small but statistical significant change in the expression of some genes in the fetal heart and liver in HIIT rats. However, there were no changes in tissue biochemistry indicative of injury by reactive oxygen species. In future studies possible long term effects of hypoxic episodes on liver, heart, central nervous system and other organs could be examined in offsprings of training animals.

## Conclusion

This study indicates HIIT is feasible and well tolerated by pregnant rats. Six weeks of HIIT did not lead to significant heart remodeling in young, female rats whether they become pregnant or not. There were no adverse effects of HIIT in pregnancy on the mother and it did not alter the structure and function of maternal heart. The fetal and placental growth was not affected. The total antioxidant capacity in the placenta and fetal heart was lower than in the liver suggesting that suggesting that the fetal liver is well protected against reactive oxygen species. Oxidative stress and total antioxidant capacity in placenta, fetal heart and liver were not affected by HIIT. However, some genes related to oxidative stress were altered in the fetal heart and liver indicating that protective mechanisms are activated. Before extensive training at the anaerobic threshold can be ruled as safe in pregnant women, clinical studies with meticulous monitoring of the fetus must be performed.

## Supporting Information

S1 File Main data set. (SAV)

S2 File Quantitative RT-PCR on maternal heart tissue, data set 1. (XLSX)

S3 File Quantitative RT-PCR on maternal heart tissue, data set 2. (XLSX)

S4 File Quantitative RT-PCR on maternal heart tissue, data set 3. (XLSX)

S5 File Quantitative RT-PCR on fetal heart tissue, data set 1. (XLSX)

S6 File Quantitative RT-PCR on fetal heart tissue, data set 2. (XLSX)

S7 File Quantitative RT-PCR on fetal liver tissue, data set 1. (XLSX)

S8 File Quantitative RT-PCR on fetal liver tissue, data set 2. (XLSX)

S9 File Quantitative RT-PCR on placentas, data set 1. (XLSX)

S10 File Quantitative RT-PCR on placentas, data set 2. (XLSX)

S11 File Peroxidase activity, superoxide dismutase activity, malondialdehyde content and total antioxidant capacity in fetal hearts, fetal livers and placentas, data set. (XLSX)

S1 Table Reference genes and primers used for real-time polymerase chain reaction analysis. (DOC)
