# Supplementary material for: Effects of High Intensity Interval Training on Pregnant Rats, and the Placenta, Heart and Liver of Their Fetuses
Source: PLoS One. 2015 Nov 13;10(11):e0143095. doi: 10.1371/journal.pone.0143095 (PMC4643918; doi:10.1371/journal.pone.0143095)
Supplement: S1 Table — (DOC) [file pone.0143095.s012.doc]

S1 Table

Reference genes and primers used for real-time polymerase chain reaction analysis.

| RAT mRNA | Primer sequence (5’-3’) |
| --- | --- |
| **Reference genes** | |
| ***GADPH: Glyceraldehyde-3phosphate dehydrogenase*** | |
| Forward primer | CTG-CAC-CAC-CAA-CTG-CTT-AC |
| Reverse primer | CAG-AGG-TGC-CAT-CCA-GAG-TT |
| ***HPRT: Hypoxanthine phosphoribosyltransferase*** | |
| Forward primer | GAC-CGG-TTC-TGT-CAT-GTC-G |
| Reverse primer | ACC-TGG-TTC-ATC-ATC-ACT-AAT-CAC |
| ***Cyclo: Cyclophilin*** | |
| Forward primer | CTG-ATG-GCG-AGC-CCT-TG |
| Reverse primer | TCT-GCT-GTC-TTT-GGA-ACT-TTG-TC |
| ***B2M: β-2 microglobulin*** | |
| Forward primer | TGC-CAT-TCA-GAA-AAC-TCC-CC |
| Reverse primer | GAG-GAA-GTT-GGG-CTT-CCC-ATT |
| ***LDHA: Lactate dehydrogenase*** | |
| Forward primer | GAT-CTC-GCG-CAC-GCT-ACT |
| Reverse primer | CAC-AAT-CAG-CTG-GTC-CTT-GAG |
| ***SDHA: Succinate dehydrogenase complex, subunit A*** | |
| Forward primer | CCC-TGA-GCA-TTG-CAG-AAT-C |
| Reverse primer | CAT-TTG-CCT-TAA-TCG-GAG-GA |
| ***RPL13a: 60S ribosomal protein L13a*** | |
| Forward primer | GAT-CTC-GCG-CAC-GCT-ACT |
| Reverse primer | GGT-ACT-TCC-ACC-CGA-CCT-C |
| **Studied genes** | |
| ***PKC-α: Protein kinase C-α*** | |
| Forward primer | CAA-GCA-GTG-CGT-GAT-CAA-TGT |
| Reverse primer | GGT-GAC-GTG-CAG-CTT-TTC-ATC |
| ***PKC-δ: Protein kinase C-δ*** | |
| Forward primer | TCA-AGA-ACC-ACG-AGT-TCA-TCG |
| Reverse primer | GCA-TTG-CCT-GCA-TTT-GTA-GC |
| ***PKC-ε: Protein kinase C-ε*** | |
| Forward primer | CGT-CAC-TGA-TGT-GTG-CAA-TG |
| Reverse primer | TCG-AAC-TGG-ATG-GTG-CAG-TTG |
| ***α-MHC: α-Myosin heavy chain*** | |
| Forward primer | CAA-GGC-AAA-CCT-GGA-GAA-AG |
| Reverse primer | GGG-TAT-AGG-AGA-GCT-TGC-CC |
| ***β-MHC: β- Myosin heavy chain*** | |
| Forward primer | GAG-GAG-AGG-GCG-GAC-ATT |
| Reverse primer | ACT-CTT-CAT-TCA-GGC-CCT-TG |
| ***ANP:*** ***Atrial natriuretic peptide*** | |
| Forward primer | CAA-CAC-AGA-TCT-GAT-GGA-TTT-CA |
| Reverse primer | CGC-TTC-ATC-GGT-CTG-CTC |
| ***BNP:*** ***B-type natriuretic peptide*** | |
| Forward primer | GTC-AGT-CGC-TTG-GGC-TGT |
| Reverse primer | CAG-AGC-TGG-GGA-AAG-AAG-AG |
| ***ANKRD1: Ankyrin repeat domain-containing protein 1*** | |
| Forward primer | GCTGGAGCCCAGATTGAA |
| Reverse primer | CTCCACGACATGCCCAGT |
| ***TNF-α:*** ***Tumor necrosis factor-α*** | |
| Forward primer | GCC-CAG-ACC-CTC-ACA-CTC |
| Reverse primer | CCA-CTC-CAG-CTG-CTC-CTC-T |
| ***TGF-β1: Transforming growth factor β1*** | |
| Forward primer | AAG-AAG-TCA-CCC-GCG-TGC-TA |
| Reverse primer | TGT-GTG-ATG-TCT-TTG-GTT-TTG-TCA |
| ***TGF-β2: Transforming growth factor β2*** | |
| Forward primer | ATC-GAT-GGC-ACC-TCC-ACA-TAT-G |
| Reverse primer | GCG-AAG-GCA-GCA-ATT-ATC-CTG |
| ***TGF-β3: Transforming growth factor β3*** | |
| Forward primer | CCC-GAT-GGC-GAA-AGG-CCG-AG |
| Reverse primer | TAG-GGT-AGC-CGG-AGG-CCC-CT |
| ***Cth: Cystathinase*** | |
| Forward primer | TGG-GAC-CAG-AGC-CGG-AGC-AA |
| Reverse primer | AAG-GCC-CCG-AGC-GAA-GGT-CA |
| ***VEGF-α: Vascular endothelial growth factor-α*** | |
| Forward primer | CAA-GCC-AAG-GCG-GTG-AGC-CA |
| Reverse primer | TCT-GCC-GGA-GTC-TCG-CCC-TC |
| ***VEGF-β: Vascular endothelial growth factor-β*** | |
| Forward primer | ACC-AGA-AGA-AAG-TGG-TGT-CAT-G |
| Reverse primer | TGA-GGA-TCT-GCA-TTC-GGA-CTT-G |
| ***SOD1: Superoxide dismutase 1*** | |
| Forward primer | TTC-GTT-TCC-TGC-GGC-GGC-TT |
| Reverse primer | TTC-AGC-ACG-CAC-ACG-GCC-TT |
| ***eNOS: Endothelial nitric oxide synthase*** | |
| Forward primer | TGA-CCC-TCA-CCG-ATA-CAA-CA |
| Reverse primer | CGG-GTG-TCT-AGA-TCC-ATG-C |
| ***iNOS: Inducible nitric oxide synthase*** | |
| Forward primer | ACCATGGAGCATCCCAAGTA |
| Reverse primer | CAGCGCATACCACTTCAGC |
| ***COL1A1: Collagen type I-α1*** | |
| Forward primer | CAT-GTT-CAG-CTT-TGT-GGA-CCT |
| Reverse primer | GCA-GCT-GAC-TTC-AGG-GAT-GT |
| ***COL3A1: Collagen type III-α1*** | |
| Forward primer | TCC-CCT-GGA-ATC-TGT-GAA-TC |
| Reverse primer | TGA-GTC-GAA-TTG-GGG-AGA-AT |
| ***FN1: Fibronectin 1*** | |
| Forward primer | CAG-CCC-CTG-ATT-GGA-GTC |
| Reverse primer | TGG-GTG-ACA-CCT-GAG-TGA-AC |
| ***TIMP1: Tissue inhibitor of metallopeptidase 1*** | |
| Forward primer | CAG-CAA-AAG-GCC-TTC-GTA-AA |
| Reverse primer | TGG-CTG-AAC-AGG-GAA-ACA-CT |
| ***SOD2: Superoxide dismutase 2*** | |
| Forward primer | ATT-AAC-GCG-CAG-ATC-ATG-CA |
| Reverse primer | CCT-CGG-TGA-CGT-TCA-GAT-TGT |
| ***TIMP 3: Tissue inhibitor of metallopeptidase 3*** | |
| Forward primer | GAA-CGG-AAG-CGT-GCA-CAT-G |
| Reverse primer | CAG-CTT-CTT-TCC-CAC-CAC-TTT-G |
| ***TIMP 4: Tissue inhibitor of metallopeptidase 4*** | |
| Forward primer | AGG-GAG-AGC-CTG-AAT-CAT-CA |
| Reverse primer | GCA-CTG-CAT-AGC-AAG-TGG-TG |
| ***HIF1A: Hypoxia-inducible factor 1α*** | |
| Forward primer | TGC-TTG-GTG-CTG-ATT-TGT-GA |
| Reverse primer | GGT-CAG-ATG-ATC-AGA-GTC-CA |
| ***CAT: Catalase*** | |
| Forward primer | TTT-TCA-CCG-ACG-AGA-TGG-CA |
| Reverse primer | CCC-ACA-AGG-TCC-CAG-TTA-CC |
| ***HK2: Hexokinase II*** | |
| Forward primer | TCG-CAT-ATG-ATC-GCC-TGC-TT |
| Reverse primer | GCC-ATT-GTC-CGT-CAC-CCT-TA |
| ***GPx1: Glutathione peroxidase 1*** | |
| Forward primer | AGT-TCG-GAC-ATC-AGG-AGA-A |
| Reverse primer | AGG-GCT-TCT-ATA-TCG-GGT-TC |
| ***GPx2: Glutathione peroxidase 2*** | |
| Forward primer | GCC-TAG-TGG-TTC-TCG-GCT-TCC |
| Reverse primer | AGG-GTA-GGG-CAG-CTT-GTC-TTT-C |
| ***GPx4.1: Glutathione peroxidase 4 transcript variant 1*** | |
| Forward primer | GCC-GCT-TAT-TGA-AGC-CAG-C |
| Reverse primer | GTG-GGC-ATC-GTC-CCC-ATT-TA |
| ***GPx4.2: Glutathione peroxidase 4 transcript variant 2*** | |
| Forward primer | CCC-ATT-CCC-GAG-CCT-TTC-AA |
| Reverse primer | TAT-CGG-GCA-TGC-AGA-TCG-AC |
| ***Ddx3y: DEAD box polypeptide 3, Y-linked*** | |
| Forward primer | ACG-GTG-GCT-TGC-TCC-GTG-AA |
| Reverse primer | GCC-AAC-CGT-ATT-TTC-CGC-CGC |
| ***Eif2s3y: eukaryotic translation initiation factor 2, subunit 3, Y-linked*** | |
| Forward primer | GGT-TGG-GCA-GGT-CCT-TGG-TGC |
| Reverse primer | CGC-CAG-TGC-TTT-TCA-ACT-CGT-CG |
